# Dr. Cox on Acute Rheumatism

**Published:** 1825-04-01

**Authors:** 


*Observations on Acute Rheumatism, and its Metastasis to the Heart, &c.* By THOMAS COX, M.D. Fellow of the Medical Society of London, &c. Octavo, pp. 65. London, 1824.

ACUTE Rheumatism is a far more dangerous disease than is generally imagined. We do not mean to say that it is often fatal, *as external rheumatism;* but we mean to affirm, from long and attentive observation, that, *in its consequences,* it is productive of a very considerable proportion of those active enlargements, or hypertrophiæ of the heart, which we now so frequently meet in practice. This appears to be the opinion of Dr. Cox, who, though a young man, has had considerable field for observation in the Borough hospitals, and other institutions of this metropolis.

"Had not many opportunities occurred to me, of witnessing the immediate and remote consequences of this disease, during a connexion of several years with Guy's Hospital, and other public institutions, I should not have ventured to take up the pen on the present occasion. The numerous cases of organic disease of the heart and pericardium, that were referable to, or connected with rheumatitis, first attracted my attention to the subject. The individuals suffering from such disease, are not only incapacitated from performing their ordinary duties with comfort to themselves, from the general derangement of the system which disease, in so important an organ, could not fail to induce; but the serious and irreparable mischief connected therewith, too frequently removes to a premature grave some of the most interesting members of society, who were, but a short time before, in the enjoyment of perfect health." *Preface* vii.

It is Dr. Cox's object to draw the attention of his brethren to such judicious modes of treating acute rheumatism (or, as he terms it, and not, we think, inappropriately, *rheumatitis*) as may prevent or lessen these deplorable sequelæ of the disease. For this purpose, after a short description of the complaint, its causes &c. he takes a brief review of the different remedies which have been employed in the cure of the affection.

I. *Venesection.* Of all acute inflammations, the rheumatic least imperiously demands blood-letting. This remedy, is carried, in general, a great deal too far, as we have had abundant opportunities of witnessing. Many practitioners think they can reduce the pain, swelling, and inflammation of rheumatic limbs, with the same facility, and by the same reiterations of venesection, as they would pleuritis. But they are generally foiled—and when they think themselves most triumphant, they have most endangered the future health and lives of their patients. By this ultra-depletion they may induce *immediate* retrocession of the disease to an internal organ, for there is no trifling relationship between rheumatism and gout—or what is more generally the case, they lay the foundation for a slow and *chronic affection* of the heart, which they afterwards consider as a new disease, totally unconnected with the rheumatitis.

Dr. Cox is an advocate for early and moderate venesection—and in this we agree with him, though we by no means consider venesection, in any degree, so essential to the cure of acute rheumatism as Dr. Cox appears to view it. The very circumstance of the disease being treated by many able physicians with bark, and successfully, proves that rheumatism is not a common phlogosis. Who ever treated successfully pneumonia or enteritis with bark?

"The inflammation, when confined to the joints, does not endanger life; consequently that promptitude of practice, so absolutely necessary in acute visceral inflammation, is not required; for we may, after the first bleeding, allow our patients to remain twenty-four or thirty-six hours with impunity. The mere existence of pain, however severe, will not always warrant a continuance of blood-letting; for it is observed that rheumatic inflammation, under every mode of treatment, will often run a certain course; it is, therefore, evident that the rash abstraction of blood would only debilitate and protract the cure." 21

II. *Purgatives.* These should seldom be of the drastic kind; nor should hypercatharsis ever be induced.

III. *Diaphoretics.* These are favourite remedies, and certainly useful ones.

"The various remedies of this class it will be needless to enumerate. The *Pulv. Ipecac. Comp.* and a combination of *Submuriate of Mercury, Opium, and Antimony,* are two excellent forms and will seldom fail; more especially if aided by the free use of *Tepid Diluents,* which are strongly recommended by Sydenham and others." 24.

IV. *Colchicum.* This is a powerful auxiliary in rheumatism. We doubt whether it is safe to quickly arrest the progress of this disease by colchicum, in large doses, where there is any weak organ internally. We apprehend that, in such cases, the same reasonings will apply as in the treatment of gout. There can be no objection, however, when metastasis of rheumatism has produced disease of an internal organ, to the moderate employment of colchicum, although it might be improper in the open and developed forms of the complaint.

At page 39, Dr. Cox comes to the subject of metastasis to the heart, and gives a rapid sketch of its history, which, as our readers well know, is only of recent date. He thinks that, "the majority of cases of organic disease of the heart in young people, are connected with rheumatism"—and perhaps the period might be extended beyond that of youth.

In respect to the post-mortem appearances and the treatment of rheumatic metastasis, Dr. Cox does not present any thing novel. Of the cases brought forward in illustration, we shall introduce the following.

"*December* 22, 1822.

"William Brown, æt. 20, was attacked four days ago, after exposure to wet and cold, with febrile symptoms, soon followed by acute pains in several of the joints, which are at present very severe, with pain in the region of the heart: respiration, hurried and anxious: palpitation considerable; countenance distressed; cough frequent, loose, and painful; pulse 120, strong, hard, and contracted; tongue white; much thirst; bowels constipated.

Sit V.S. ad ℥xvj statim.

Cap. Inf. Sennæ et Magn. Sulph. 2da q.q.

h. donec alv. purg. et postea

Tinct. Digit. ♏ x 4ta q.q. h. ex Inf. Rosæ.

"23d.—No relief by bleeding; blood buffed; symptoms nearly the same:

Rep. V.S. ad ℥xij, et Digit. ut antea.

"24th.—Bleeding gave temporary relief only to pain of chest; joints somewhat easier; pulse 126, jirking.

Rep. Digit.

℞ Hydr. Submur. gr. iij

Antim. Tartar. gr. $\frac{1}{3}$

Opii gr. j. ft. Pil. h. s. s.

Cuc. Cr. ad ℥x reg. cordis.

"25th.—Pain in left side of chest less acute; anxiety of countenance and distress in breathing the same; makes but little complaint of joints; pulse as yesterday.

Appr. Hirudines. xij lat. sinist. et Emp.
Lyttæ postea.
Intermitt. Med.
Sumt. Ext. Conii gr. v. c Ipecac. gr. j
8° ter die.

"This lad lived till Jan. 8th, without any abatement to his sufferings, and towards the close the anxiety of countenance and distress in breathing were considerable.

"*Dissection.*—A honey comb deposit on the heart and inner surface of the pericardium, with a few bands of adhesive matter attaching these parts together: a few ounces of very turbid serum were contained in the pericardium, with flakes of lymph floating in it. Heart itself not enlarged nor altered in structure." 57.

The following is a case of metastasis to the brain—a translation not so common as to other parts of the body.

"Mr. S. æt. 25, after exposure to cold, in October last was attacked with acute Rheumatism. The pains of the ancles and wrists were so exceedingly distressing as to induce the surgeon to apply leeches, the result of which was almost immediate relief; at the same time he took a brisk purgative, and subsequently diaphoretics.

"About twenty-four hours after the application of the leeches, I first saw this gentleman, at which time he complained much of a sense of weight in the head, and some impatience of light and sound, accompanied with a wild anxious expression of countenance, occasional rambling, strabismus, and impaired vision; pulse 110, hard and full; tongue whitish and moist; skin soft, with some perspiration.

"From the history of this patient, more particularly in reference to the mode of treatment, I was disposed to consider it a case of metastasis to the brain, although the mental faculties were unimpaired, and no decided delirium accompanied it.

"With this persuasion, I proposed a full bleeding from the arm (sixteen ounces,) and the exhibition of five grains of submuriate of mercury, succeeded immediately by senna and salts, until several copious evacuations were produced. We also agreed on the application of leeches to the temples, and cold lotion to the shaved head, in the evening, provided the symptoms did not materially subside.

"The symptoms continuing at night, the measures agreed upon were pursued.

"On the following morning, the only alteration in the symptoms was a diminution in the impatience of light and sound. The blood drawn yesterday was much buffed and cupped.

" A repetition of venesection, to ℥xij, and purgative medicines were prescribed, with a perseverance in the cold lotion.

" In the evening, the bleeding was repeated to ℥x; from the highly inflammatory character of the blood drawn in the morning, and from symptoms remaining which indicated that mischief was still going on. On the next (third) day, the bleeding was repeated to twelve ounces, and eight ounces were drawn on the fifth day.

" The blood, to the last, exhibited signs of inflammation; after this, by keeping up a constant action on the bowels, the head affection was gradually subdued, and on the 8th day from its appearance had entirely subsided." 61.

The following is an example of pericarditis.

" Thomas Cope, æt. 27, had laboured under a severe stricture of the urethra, with fistulous openings in the perineum, for several years, which had been much relieved by the use of sounds, &c. afterwards an operation was performed for the cure of the fistulæ, Nov. 27, 1823, the result of which was in every respect satisfactory. On Dec. 19, he was suddenly attacked with pain in the epigastrium, increased by pressure and a full inspiration; pulse 90, rather oppressed and contracted, accompanied with an anxious expression of countenance: hurried respiration, and occasional vomiting; tongue dry, and red in centre; bowels open.

Sit. V.S. ad ℥xij statim.

℞ Hydr. Submur. gr. j.
Opii Extract. gr. j ft.
Pil. 6ta q.q. h. sumend.

" 20th.—Blood much buffed and cupped; tongue universally brown and dry; pulse 100, hard and contracted; makes very little complaint of pain; anxiety of countenance increased; almost constant nausea.

Rep. V.S. ad ℥xij.

" On the evening of the 21st, there was considerable intermission and irregularity in the pulse, and he was in every respect much worse, and decidedly sinking.

" 23d.—Died early this morning.

" *Dissection.*—The investing and reflected portions of the pericardium were covered with adhesive matter, and flakes of lymph floated in about three ounces of turbid serum contained in the pericardium; substance of the heart healthy; no valvular disease; *liver* much indurated and granular, and it had contracted adhesions to the neighbouring parts, which were firm, and consequently of long standing." 65.

Dr. Cox's observations are generally judicious, but the work presents no novel or striking views of the etiology, pathology, or treatment, of the disease forming the subject of investigation.

